# Piloting the first health system performance assessment for Germany: key results and learnings

**DOI:** 10.1093/eurpub/ckac129.245

**Published:** 2022-10-25

**Authors:** M Blümel, M Achstetter, P Hengel, M Schwarzbach, R Busse

**Affiliations:** Berlin University of Technology, Department of Healthcare Management, Berlin, Germany

## Abstract

**Background:**

Health System Performance Assessment (HSPA) is a tool to monitor and evaluate the performance of health systems and to inform evidence-based policymaking. For the first time, a country specific HSPA is currently being piloted for Germany.

**Methods:**

The HSPA is based on a newly developed conceptual framework including nine dimensions (e.g., access, quality, efficiency, population health). Indicators were selected based on a systematic search of (inter)national studies and HSPA initiatives. Where possible, indicators were analysed in their development over time (2000-2020), in comparison to eight European countries (e.g., Austria, Denmark, France), and along up to seven equity dimensions (e.g., sex, age, income, education, region).

**Results:**

Overall, 90 indicators were included in the HSPA. Trend and equity analyses were possible for almost all and country comparisons for most indicators. A few indicators could not be analysed at all due to missing data. The overall HSPA provides an in-detail picture of Germany's health system. Access, for example, can be rated as good in Germany compared to the other countries, as insurance coverage and physician density are high, and unmet needs and waiting times for elective surgery are low. Results for quality are not as good, e.g., cancer survival rates, but most indicators show a positive trend. While population health outcomes are average in country comparison (e.g., fetal and infant mortality), resource input is comparatively high. Consequently, overall efficiency can still be improved (e.g., amenable mortality per total health expenditure).

**Conclusions:**

This first HSPA for Germany allows new insights to the performance of the German health system which are important for policy and research. While the pilot benefitted a lot from previous HSPA initiatives, data availability remains one of the biggest challenges.

